# Vitamin D supplementation and body composition changes in collegiate basketball players: a 12-week randomized control trial

**DOI:** 10.1080/15502783.2022.2046444

**Published:** 2022-03-22

**Authors:** Tamara Hew-Butler, Carrie Aprik, Brigid Byrd, Jordan Sabourin, Matthew VanSumeren, Valerie Smith-Hale, Andrew Blow

**Affiliations:** a Exercise Science and Athletics Departments, Oakland University, Rochester, Michigan, USA; b Division of Kinesiology, Health and Sport Studies, Wayne State University, Detroit, Michigan, USA; c Precision, Fuel & Hydration, Minneapolis, Mn, USA

**Keywords:** 25-hydroxyvitamin D, basketball, body composition, sweat sodium

## Abstract

**Background:**

Vitamin D promotes bone and muscle growth in non-athletes, suggesting supplementation may be ergogenic in athletes. Our primary aim was to determine if modest Vitamin D supplementation augments favorable body composition changes (increased bone and lean mass and decreased fat mass) and performance in collegiate basketball players following 12 weeks of standardized training.

**Methods:**

Members of a men’s and women’s NCAA D1 Basketball team were recruited. Volunteers were randomized to receive either a weekly 4000 IU Vitamin D_3_ supplement (D3) or placebo (P) over 12 weeks of standardized pre-season strength training. Pre- and post-measurements included 1) serum 25-hydroxy vitamin D (25(OH)D); 2) body composition variables (total body lean, fat, and bone mass) using dual-energy X-ray absorptiometry (DXA) scans and 3) vertical jump test to assess peak power output. Dietary intake was assessed using Food Frequency questionnaires. Main outcome measures included changes (∆: post-intervention minus pre-intervention) in 25(OH)D, body composition, and performance.

**Results:**

Eighteen of the 23 players completed the trial (8 females/10 males). Eight received the placebo (20 ± 1 years; 3 females) while ten received Vitamin D_3_ (20 ± 2 years; 5 females). Weekly Vitamin D_3_ supplementation induced non-significant increases (∆) in 25(OH)D (2.6 ± 7.2 vs. −3.5 ± 5.3 ng/mL; p = 0.06), total body bone mineral content (BMC) (73.1 ± 62.5 vs. 84.1 ± 46.5 g; p = 0.68), and total body lean mass (2803.9 ± 1655.4 vs. 4474.5 ± 11,389.8 g; p = 0.03), plus a non-significant change in body fat (−0.5 ± 0.8 vs. −1.1 ± 1.2%; p = 0.19) (Vitamin D_3_ vs. placebo supplementation groups, respectively). Pre 25(OH)D correlated with both Δ total fat mass (g) (r = 0.65; p = 0.003) and Δ total body fat% (r = 0.56; p = 0.02). No differences were noted in peak power output ∆ between the D3 vs. P group (−127.4 ± 335.4 vs. 50.9 ± 9 W; NS). Participants in the D3 group ingested significantly fewer total calories (−526.2 ± 583.9 vs. −10.0 ± 400 kcals; p = 0.02) than participants in the P group.

**Conclusions:**

Modest (~517 IU/day) Vitamin D_3_ supplementation did not enhance favorable changes in total body composition or performance, over 3 months of training, in collegiate basketball players. Weight training provides a robust training stimulus for bone and lean mass accrual, which likely predominates over isolated supplement use with adequate caloric intakes.

## 1. Introduction

Vitamin D supplementation is widely touted as an ergogenic aid [1]. The enhancement of performance appears to be most pronounced in athletes known to be Vitamin D-deficient prior to supplementation [2]. While the performance-enhancing benefits of Vitamin D are widely debated, the consequences of Vitamin D (routinely measured as serum 25(OH)D) deficiency (<20 ng/mL) and insufficiency (20–29 ng/mL) are well described in the general population [3].

Athletes with Vitamin D deficiency have smaller hearts [4], have decreased lean mass [5], and are at greater risk for bone fractures [6] as well as frequent illness [7] when compared to peers with higher serum Vitamin D levels. Basketball players [8] and African American athletes [1] appear to be at greatest risk for Vitamin D deficiency presumably due to differences in both diet and darkened skin complexion. However, a recent study suggests that African Americans have lower amounts of Vitamin D-binding protein, so that a greater proportion of bioactive vitamin is available, despite lower 25(OH)D levels [9].

A systematic review and meta-analyses of 15 studies suggests that 77% of basketball players are 25(OH)D insufficient [10], while cross-sectional studies confirm that 94% of adolescent basketball players are Vitamin D insufficient [8] while 57% of Spanish professional basketball players are Vitamin D deficient [1]. An investigation performed on 11 National Collegiate Association Athletes (NCAA) Division 1 (D1) basketball players demonstrated a 6.1% decrease in total bone mineral content (BMC) from pre-season to late summer, which was reversed the following year when a robust calcium supplement (~2000 mg/day), coupled with a standardized 400 IU Vitamin D supplement, was introduced to preserve bone mass [11]. Our prior data identified the men’s basketball team as having 25(OH)D concentrations, which classified them as insufficient pre-season (21.8 ± 7.9ng/L) and deficient post-season (14.5 ± 5.6 ng/mL) [12]. Collectively, with the (recommended) threshold for ‘peak performance’ set at >50 ng/mL [2] plus the University’s exposure to long Vitamin D deficient winters (latitude 42°) [13], our basketball players appeared at greatest risk for bone injuries due to Vitamin D deficiency.

The *primary* purpose of this pilot study was to investigate the effects of modest (4000 IU/week, equivalent to 517IU/day) Vitamin D_3_ supplementation on bone and body composition changes in collegiate basketball players during 3 months of organized summer strength training. A *secondary* purpose was to assess potential body composition differences between African American and Caucasian players. *Tertiary* purposes (from secondary analyses) investigated performance and other factors, which potentially influence total body bone mass (i.e. dietary intakes, sweat sodium loss, lean, and/or fat mass).

## 2. Materials and methods

After Institutional Review Board (IRB) approval was granted (IRB#922734), all members of a men and women’s NCAA D1 basketball team were recruited to participate. All subjects gave informed consent before participating in the study, which was conducted in accordance with the Declaration of Helsinki. The study protocol was registered (retroactively) as identifier International Standard Randomized Controlled Trials Number (ISRCTN) #14,155,111.

After obtaining written informed consent, all participants underwent pre-intervention (baseline) testing, which was conducted in July (prior to summer strength training). Players were then randomized (single-blind, with participants blinded to the intervention) to receive either a Vitamin D (Vitamin D_3_, 4000 IU soft gels, Up&Up™, Greenville, SC) or a placebo capsule (Gelatin 1300 mg Spring Valley, Bentonville, AR) weekly. The randomization of subjects utilized an alternating (odd and even) number distribution, while ensuring that a racial balance between African American and Caucasian players existed between the two supplement groups, during the randomization process.

We chose to supplement weekly for logistical reasons (i.e. the IRB limited the daily dose to 4000 IU, and we were only allowed access the basketball players once per week), with the dosage based upon current recommendations (400–600 IU/day with a daily tolerable upper limit of 4000 IU) for healthy young (19–50 years) individuals [3]. Weekly supplementation was supervised by the research team over a 12-week period. Researchers brought the supplements in (labeled) plastic cups to the weight-training room and dispensed them after completion of Monday’s workout. Participants ingested the supplements with milk *ad libitum* and completed a weekly questionnaire estimating how much time each player spent in the sun, between the hours of 10 am and 3 pm. Post-intervention testing was conducted in October, just prior to the start of official basketball practice. The following tests were performed pre- and post-intervention.

### 2.1. *For* primary and secondary outcomes

Blood Measures – 25(OH)D was assessed by a local hospital laboratory (Cobas E Immunoassay, Ascension-Crittenton Hospital, Rochester, Michigan) following venipuncture in a supine position.

Body composition *–* After a height and weight measurement was obtained on a weight beam scale, total body lean, fat, and bone mass were assessed using a whole-body dual-energy x-ray absorptiometry (DXA) scan (Horizon A, software version 13.6.0.5, Hologic®, Bedford, MA). All DXA scans were performed by a single technician following standardized protocols, based on the product user manual [14].

### 2.2. *For* tertiary outcomes

Dietary Evaluation – Dietary intake was assessed using the Block 2005 3-month Food Frequency questionnaire (NutritionQuest, Berkeley, CA), completed under laboratory supervision at the time of testing. Dietary measures of specific interest included total calorie, Vitamin D, calcium, and sodium intake.

Sweat Testing – At pre-intervention testing only, sweat sodium concentration was assessed on the non-dominant forearm (volar surface, free of tattoos) via pilocarpine iontophoresis as described in detail elsewhere [15].

Performance – A standing vertical jump test was performed, using a vertec. The highest of three attempts was recorded and converted to Peak Power output using the following equation: *peak power (watts) = 60.7 × (jump height [cm]) + 45.3 × (body mass [kg]) – 2055.*

Statistics – A repeated-measures ANOVA was used to assess significant differences between Groups (Vitamin D_3_ vs. placebo) over Time (pre-intervention vs. post-intervention). Unpaired t-tests were used to compare males vs. females, African American vs. Caucasian players, and between the change (Δ: post-intervention minus pre-intervention) variables. Simple (Pearson’s r) regression correlations were used to assess relationships between variables, with statistical significance set at p < 0.05. All data were presented as mean ± SD.

## 3. Results

**Figure 1.**
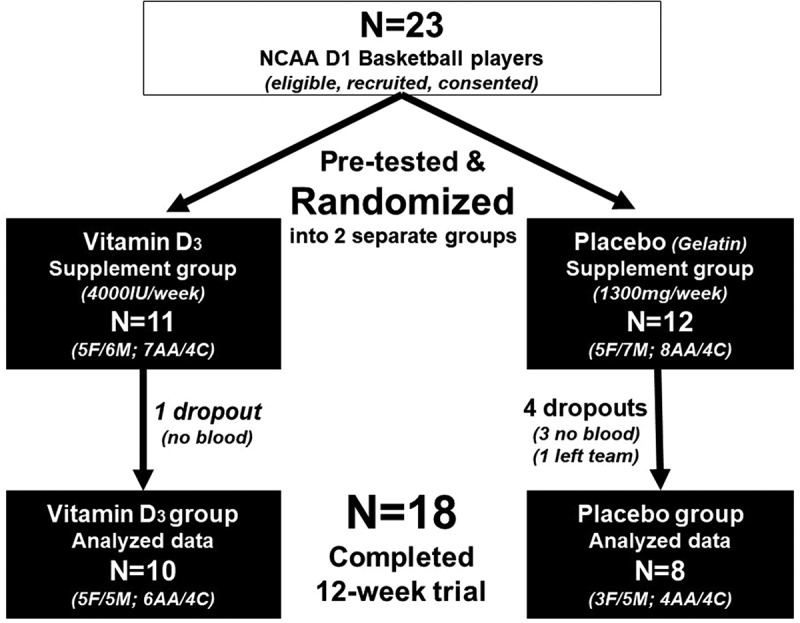
Study participant enrollment and randomization into vitamin D3 and placebo supplementation groups.

Twenty-three collegiate basketball players signed written informed consent and participated in pre-intervention testing. Five players (two females) did not complete post-testing: one was injured, two were unable to donate blood, and two did not return (one left the team). Thus, 18 players completed the trial with a full data set, including 10 males and 8 females (Figure 1).

No significant (p > 0.05) differences were noted between males and females for body mass index (BMI), 25(OH)D (pre, post, or Δ), body composition Δ’s in fat, lean, or bone mass nor dietary intake (pre, post, or Δ) for Vitamin D, calcium, sodium, or total calories. Therefore, male and female data were combined for analyses.

### 3.1. Primary outcomes

**Figure 2.**
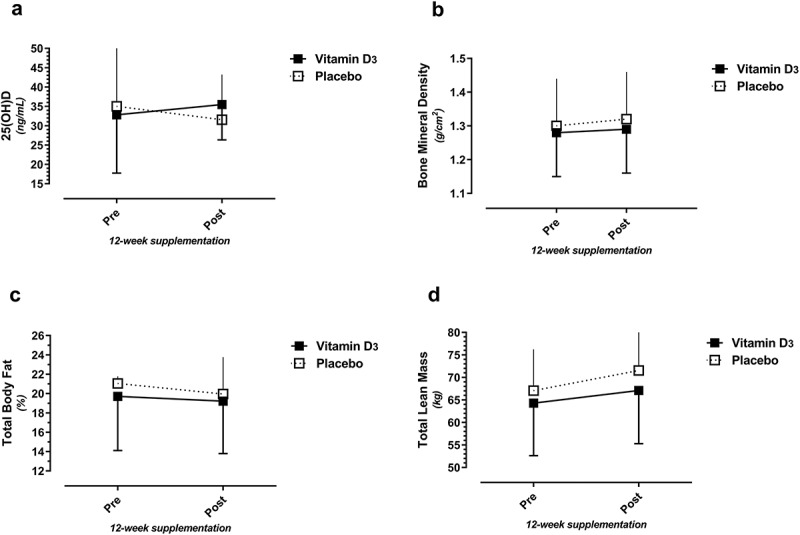
Vitamin D_3_ and placebo supplement group comparisons over time (pre- to post-intervention) for 25(OH)D (2a), bone mineral density (BMD)(2b), total body fat percent (2c), and total body lean mass (2d).

Ten players received Vitamin D_3_ (five females) while eight received the placebo (three females) supplementation. 25(OH)D levels increased in the Vitamin D supplement group (32.8 ng/mL pre to 35.5 ng/ml post) and decreased in the placebo group (35.0 ng/mL pre to 31.5 ng/mL post), but the pre- to post-intervention Δ was not statistically significant (2.6 vs. −3.5 ng/mL; p = 0.06, Vitamin D_3_ vs. placebo supplement groups, respectively). No significant differences were noted for any body composition variable (Figure 2). There were trends for participants in both supplement groups to increase total lean and bone mass and decrease fat mass, over 12 weeks of standardized group strength training.

Demographic, peak power, and dietary intake variables are detailed in Table 1, with players in the Vitamin D_3_ supplement group ingesting fewer total calories and sodium than those players receiving the placebo supplementation, over the 12-week trial period.

**Table 1.** Demographics, peak power, and dietary intake variables in basketball players randomized into the vitamin D supplement and placebo groups. F = female athletes. Δ = post minus pre intervention

VARIABLE	VITAMIN D_3_ GROUP *(n = 10; 5 F)*	PLACEBO GROUP *(n = 8; 3 F)*
Age *(years)*	20 ± 2	20 ± 1
Height *(m)*	1.83 ± 0.1	1.87 ± 0.1
Pre-Weight *(kg)*	84 ± 13	89 ± 12
Δ Weight *(kg)*	0.9 ± 1.7	2.4 ± 2.2
Pre-Peak Power *(W)*	5889.9 ± 1307.5	5997.5 ± 1016.8
Δ Peak Power *(W)*	−127.4 ± 335.4	50.9 ± 209.2
Pre-Vitamin D Intake *(IU)*	232.9 ± 162.9	242.9 ± 183.5
Δ Vitamin D Intake *(IU)*	31.3 ± 190.2	63.6 ± 205.9
Pre-Calcium Intake *(mg)*	944.2 ± 541.5	1026.5 ± 310.1
Δ Calcium Intake *(mg)*	25.5 ± 453.7	−71.9 ± 333.4
Pre-Sodium Intake *(mg)*	3469.4 ± 1850.1	2980.4 ± 797.4
Δ Sodium Intake *(mg)*	−991.4 ± 986.1**	174.1 ± 573.6
Pre-Caloric Intake *(kcals)*	2117.8 ± 1128.3	1900.2 ± 470.9
Δ Caloric Intake *(kcals)*	−526.2 ± 583.9*	−10.0 ± 400.0

*p < 0.05; **p < 0.01; ***p < 0.001 between vitamin D vs. placebo supplement groups.

**Figure 3.**
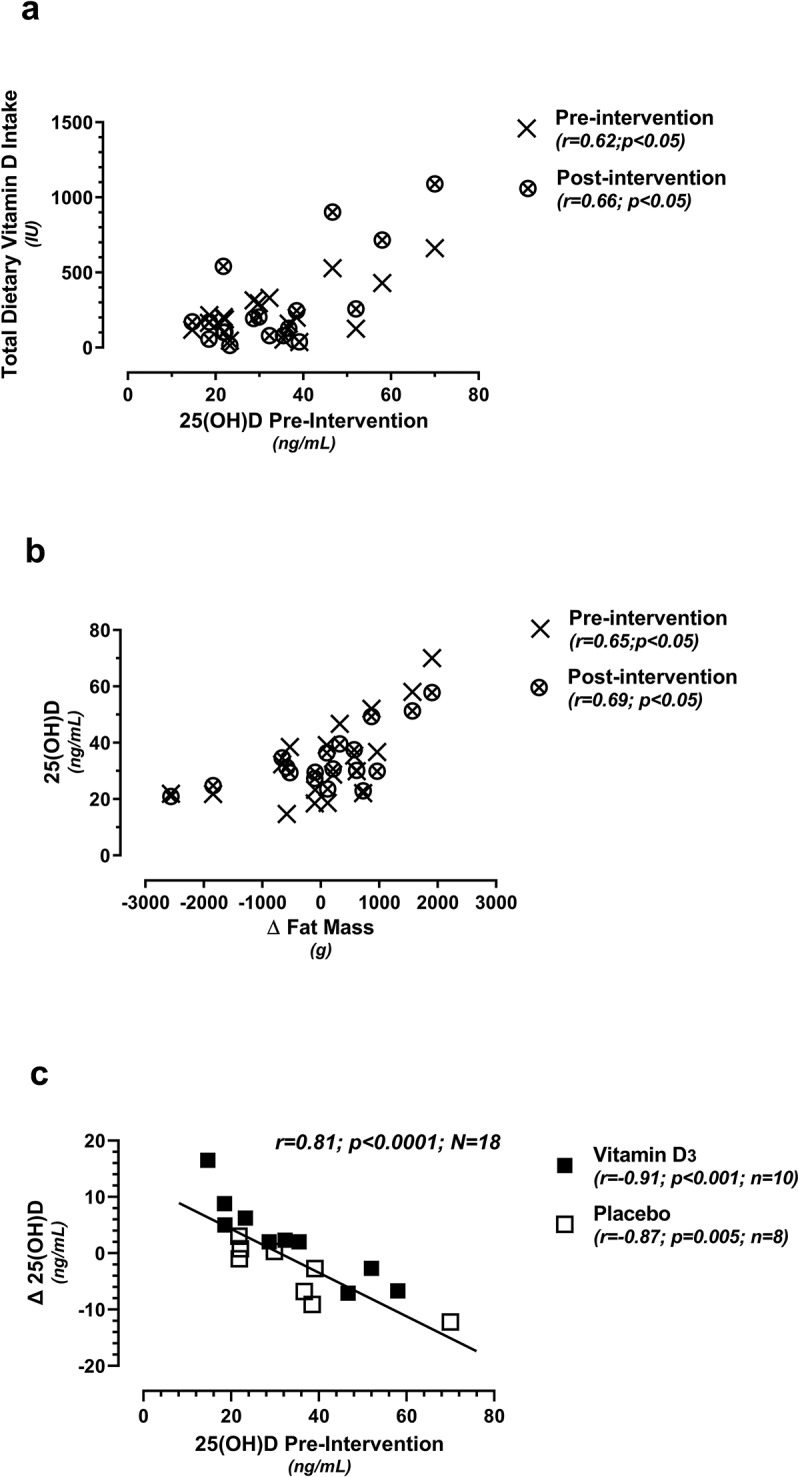
Relationships between total dietary vitamin D_3_ intake vs. 25(OH)D (3a; pre- and post-intervention); 25(OH)D vs. the change (Δ) in fat mass (3b; pre- and post-intervention); and the change (Δ) in 25(OH)D vs. pre-intervention 25(OH)D levels (3c; randomized into the placebo or vitamin D_3_ supplement group). All change (Δ) values represent post-intervention minus pre-intervention.

Pre- and post-intervention total dietary Vitamin D intake (not including supplementation), obtained from the Food Frequency questionnaire, were positively related to pre-intervention 25(OH)D (Figure 3a). Additionally, pre- and post-intervention 25(OH)D levels were positively related to changes (post minus pre) in total fat mass (Figure 3b), whereas those athletes with the highest 25(OH)D levels had the greatest pre-to-post increases in fat mass. A significant negative relationship was noted between the change (post minus pre) in 25(OH)D vs. pre-intervention 25(OH)D, as a total cohort (Figure 3c), and also when subdivided into Vitamin D_3_ and placebo supplement groups.

### 3.2. Secondary outcomes

Ten players were African American (four females) while eight were Caucasian (four females). The African American players had lower baseline 25(OH)D levels. The African American players lost more total body fat (%) and ingested more total calories (but less calcium and sodium) when compared with the changes (Δ) seen in Caucasian players (Table 2). A significant increase in the ratio of lean mass Δ over total mass Δ was seen in African American versus Caucasian players. More simply stated, African American players demonstrated increases in lean mass that were 141% greater than the increase in total mass because of concomitant fat mass losses. The Caucasian players gained both lean *and* fat mass so that on average, ~75% of the total mass gained was derived from lean mass (Table 2). Male African American players reported spending significantly less time in the sun vs. Caucasian players over the 12-week intervention period (7 ± 2 vs. 11 ± 4 hours/week; p = 0.03, respectively). Female self-reported sun exposure was incomplete, so these data were not analyzed.

**Table 2.** Differences in 25(OH)D, body composition, and dietary intake between African American and Caucasian players. F = female athletes. Δ = post minus pre-intervention

VARIABLE	AFRICAN AMERICANS *(n = 10; 4 F)*	CAUCASIANS *(n = 8; 4 F)*
Age *(years)*	20.0 ± 1.3	20.1 ± 1.6
Height *(m)*	1.83 ± 0.1	1.86 ± 0.1
Pre-Weight *(kg)*	85.8 ± 12.4	86.4 ± 13.0
Pre-25(OH)D *(I/U)*	25.8 ± 10.1**	43.7 ± 14.7
Δ 25(OH)D *(I/U)*	2.5 ± 7.4	−3.4 ± 5.0
Pre-Total Body Fat *(%)*	20.5 ± 5.3	20.2 ± 4.3
Δ Total Body Fat *(%)*	−1.3 ± 0.8**	0.0 ± 0.7
Pre-Total Lean Mass *(kg)*	65.0 ± 10.5	66.2 ± 11.0
Δ Total Lean Mass *(kg)*	3.8 ± 1.6	3.2 ± 1.9
Δ Ratio Lean/Total Mass *(%)*	141.0 ± 76.7*	75.4 ± 12.1
Pre-Total BMD *(g/cm^2^)*	1.33 ± 0.14	1.24 ± 0.10
Δ Total BMD *(g/cm^2^)*	0.01 ± 0.02	0.01 ± 0.02
Pre-Vitamin D Intake *(IU)*	219.6 ± 130.4	259.5 ± 212.0
Δ Vitamin D Intake*(IU)*	18.9 ± 198.3	79.1 ± 191.6
Pre-Calcium Intake *(mg)*	1040.9 ± 493.3	992.6 ± 565.3
Δ Calcium Intake *(mg)*	−186.4 ± 309.4*	193.1 ± 409.0
Pre-Sodium Intake *(mg)*	3526.9 ± 1427.8	2908.6 ± 1524.8
Δ Sodium Intake *(mg)*	−940.5 ± 987.9*	110.5 ± 696.9
Pre-Caloric Intake (kcals)	2193.0 ± 864.3	1806.3 ± 39.9
Δ Caloric Intake *(kcals)*	565.3 ± 516.1*	38.8 ± 440.3

*p < 0.05; **p < 0.01; ***p < 0.001 between the African American vs. Caucasian players.

### 3.3 Tertiary outcomes

**Figure 4.**
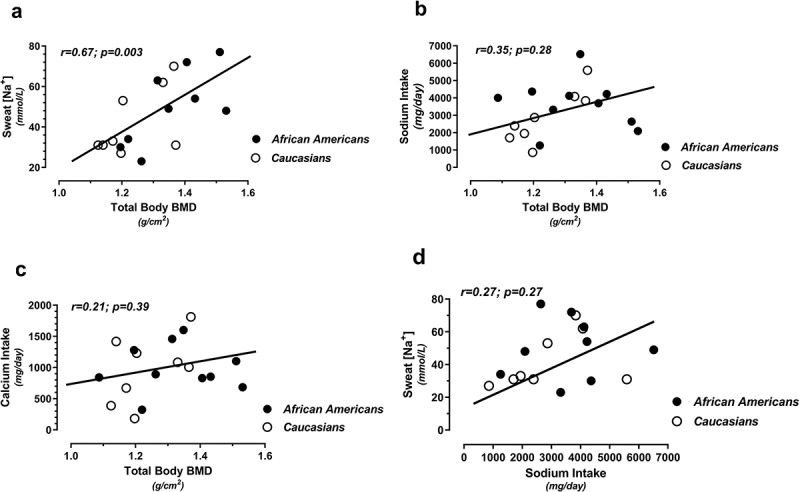
Relationships between total body bone mineral density (BMD) vs. sweat [Na^+^] (a), total dietary sodium intake (b), and total dietary calcium intake (c), and between sweat [Na^+^] vs. total dietary sodium intake (d) obtained at pre-intervention (baseline) testing.

Total body BMC was significantly correlated with total body lean mass (r = 0.93; p < 0.0001) and sweat [Na^+^] at pre-intervention (baseline) testing (r = 0.67; p = 0.003). Total body BMD was also positively related with sweat [Na^+^] (Figure 4a), with non-significant linear trends for positive relationships between total dietary sodium (Figure 4b) and calcium (Figure 4c) intake vs. total body BMD and between dietary sodium intake vs. sweat [Na^+^] (Figure 4d); all measured pre-intervention. There were no significant differences in peak power (i.e. performance) between the Vitamin D_3_ versus placebo supplement groups.

## 4. Discussion

### 4.1. Primary outcomes

Modest (~517IU/day) Vitamin D_3_ supplementation, approximating the daily dosage (400–600 IU/day) recommended for 19- to 50-year olds [3], did not enhance favorable body composition changes (i.e. increased bone and lean mass, with decreased fat mass) in basketball players following 3 months of strength training compared with placebo supplementation. One possible confounding variable in the present study, with regard to the lack of enhanced bone and lean mass gains, was the unexpected (pre-to-post) decrease in total caloric and sodium intakes seen in the Vitamin D_3_ supplement versus the placebo group (Table 1). The decrease in calories (to meet caloric deficits from heavy training) and mineral ions (sodium ions osmotically attract water into tissues) may have attenuated bone and muscle tissue gains in the Vitamin D_3_ supplement group, over the placebo group. Unfortunately, we were not able to control dietary intake (which was assessed using subjective recall), which may have further limited our ability to detect meaningful changes in body composition in our small cohort.

Of particular note, the present study demonstrated a linear (rather than dose-dependent) relationship across supplement groups during the intervention period, based upon baseline 25(OH) levels. As such, the athlete with the lowest baseline 25(OH)D level (14.7 ng/mL) responded most favorably to modest Vitamin D_3_ supplementation (increasing 25(OH)D levels by 16.5 ng/mL; Figure 3c) and vice versa. Curiously, this linear relationship between 25(OH)D change versus pre-intervention 25(OH)D was significant in the placebo as well as the Vitamin D_3_ supplemented group, which requires further clarification. A similar relationship has been previously documented in a cohort of club-level athletes (57% of which were Vitamin D deficient) receiving either 20,000 or 40,000 IU/week for 12 weeks [16]. Thus, when assessing the clinical efficacy of Vitamin D_3_ supplementation in athletes, consideration of individual needs plus baseline levels are recommended to maximize athlete health benefits [12].

Additionally, although most randomized control trials involving Vitamin D supplementation are conducted over a 12-week-period (like the present study) [^16–19^], increases in 25(OH)D have been noted after only 8 days [17] and appear to peak after 6 weeks [16] depending on the dosage. However, as in the present study, despite the achievement of sufficient 25(OH)D (>30 ng/mL) levels with vitamin D_3_ supplementation, parallel increases in physical performance [^16–19^], body composition [19], resting metabolic rate [19], and/or muscular strength [17] have yet to be documented. Consideration of seasonality [18] and length of follow-up [18] should be considered in future vitamin D_3_ randomized control trials assessing performance or body composition.

With specific emphasis on bone mass, the present study mirrored the (non-significant) results obtained in a 6-month randomized control trial performed on 32 collegiate swimmers and divers [20]. One of the stated limitations of the prior supplementation trial was that most of those participants were white (91%) and none were vitamin D deficient [20]. We thereby performed the present randomized control supplement trial on an athletic population known to be vitamin D deficient from past experience and prior studies [1; 8]. At baseline (pre-intervention), half (n = 9) of our cohort was either Vitamin D insufficient (<30 ng/mL; n = 7) or deficient (<20 ng/mL; n = 2), whereas in the swimmer study, the average 25-OH-vitamin D level across the 6-month testing period remained above 50 ng/mL [20].

In addition, a large cross-sectional study performed on 604 racially diverse male athletes identified 58.6% of participants as vitamin D deficient (<20 ng/mL). Similarly, no positive correlation was noted between 25(OH)D levels versus BMD in that study [21]. After adjusting for age and ethnicity, however, calculated bioavailable vitamin D was positively associated with hip, femoral neck, and lumbar spine BMD [21]. These authors concluded that bioavailable vitamin D (which requires measurement of vitamin D binding protein) was a more accurate predictor of bone mass than 25(OH)D levels [21]. Thus, the lack of relationship (between bone mass vs. 25(OH)D) seen in our study may also reflect a limitation in measurement, as vitamin D-binding protein was not quantified in the present study.

It could also be argued that a lack of concomitant calcium supplementation may have contributed to negligible differences in bone mass between our vitamin D_3_ versus placebo supplement groups. A prior, prospective, study performed on 11 NCAA D1 basketball players demonstrated an increase in total BMC with individualized dosing of calcium (plus a standard 400 IU/day vitamin D supplement) based upon BMC and estimated dermal (sweat) calcium losses [11]. We argue, however, that the average dietary intake of calcium in our participants hovered around ~1000 mg/day (Table 1), in addition to the milk they drank *ad libitum* with each weekly supplement. This average dietary calcium intake was well within the range of 800–1000 mg/day, as recommended by the Institute of Medicine (IOM) [22]. Thus, we do not believe that the absence of additional calcium supplementation confounded our results. We interpret our overall favorable bone mass changes (seen in both the vitamin D and placebo supplement groups) more reflective of the robust training stimulus [23] that predominated over small changes in circulating vitamin D levels.

With respect to lean and fat mass changes in our cohort, total body lean mass increased while fat mass decreased (regardless of supplement group) in response to training. Our findings contrast with a similar randomized control trial, which suggests that vitamin D supplementation decreases biomarkers for skeletal muscle breakdown (myoglobin, creatine kinase) after ultramarathons [24] and eccentric exercise [25].

Fat mass changes, in our cohort, were positively associated with 25(OH)D levels, so that increased fat mass augmented circulating vitamin D levels both pre- and post-intervention. Previous studies performed on basketball players [26], athletes [7], and non-athletes [27] demonstrated a tendency toward a negative relationship between 25(OH)D and fat mass. Accordingly, it is widely thought that adipose tissue sequesters vitamin D, with an impaired release of vitamin D in obese individuals compared with normal weight controls [28]. The present data may extend these findings to suggest that the capacity of adipose tissue to release stored vitamin D into the circulation may be enhanced in very lean and/or athletic individuals along this same continuum.

### 4.2. Secondary outcomes

As expected, our African American players demonstrated significantly lower baseline 25(OH)D levels compared with Caucasian players (Table 2), as verified in other basketball player cohorts [29,30]. Our African American players also lost significantly more body fat (%) despite a significant increase in total calories. These findings emphasize the importance of adequate caloric intake to sustain favorable anabolic effects of strength training, which are likely independent of ethnicity. Why African American players seem to adapt more favorably (i.e. higher lean mass to fat mass ratio) to a standardized strength training program remains unclear and warrants further investigation.

An unexpected racial difference, however, was our finding that male African American players spent significantly less time in the sun than male Caucasian players. Leisure time in the sun during the summer [20] and use of tanning beds in the spring [7] have been previously (and positively) associated with 25(OH)D levels, although another study conducted on basketball players demonstrated no correlation between sun exposure and 25(OH)D levels [30]. The biological explanation suggests that African Americans have less Vitamin D-binding protein (VDBP) and equal biologically active vitamin D levels as white Americans [9; 21]. However, our data support the possibility of a novel *behavioral* component to sustained low 25(OH)D levels in African American athletes living in the Midwest, worthy of further examination from a cultural perspective (i.e. anecdotally, our African American players strongly preferred remaining indoors, even during summer).

### 4.3. Tertiary outcomes

Bone mass was our main (body composition) tissue of interest, and like other randomized control trials, we did not see an augmentation of bone mass with Vitamin D_3_ supplementation. We thereby performed preliminary analyses on mineral ions to assess other possible influencers of bone mass. As such, previous research has suggested that under-replaced sweat calcium losses may contribute to the paradoxical osteopenia seen in runners [31; 32], cyclists [33], and basketball players [1] over a competitive season. Sweat calcium losses, however, have yet to be associated with changes in BMD or bone resorption markers as expected [34]. Alternatively, interest in sweat sodium (rather than calcium) losses as a contributor to athletic osteopenia has been similarly hypothesized [35]. With ~30% of total body sodium located within the skeleton [36] and osteopenia associated with chronic hyponatremia in humans [37; 38], it is tempting to speculate that consistent sweat [Na^+^] losses over time may contribute to decreased bone mass in athletes. We thereby tested this hypothesis by measuring the sodium content in pharmacologically stimulated sweat during pre-intervention testing. Paradoxically, resting sweat sodium concentrations ([Na^+^]) were positively associated with both total body BMC and BMD (Figure 4a). We hypothesized that the positive relationship between sweat [Na^+^] and total bone mineral content represented an additional (extrarenal) regulatory route for sodium secretion/excretion, in response to increased sodium ingestion when the capacity for skeletal sodium storage has been maximized. Accordingly, the estimated dietary sodium intake of our collective cohort averaged >3000 mg/day (Tables 1 and 2), which was well above the IOM recommendations of 1500–2300 mg/day [39]. There were tendencies for total body BMC to be related to self-reported dietary intakes of sodium and calcium (Figure 4b and 4c) and between sweat [Na^+^] vs. sodium intake (Figure 4d) (as demonstrated elsewhere [40]), which would trend toward supporting this hypothesis in larger cohorts. These trends encourage further exploration of the complex interplay between sodium, calcium, bone metabolism, and vitamin D, which have been proposed but under-investigated [41; 42].

Of final note, two male athletes, allocated into the vitamin D supplement group during summer training, sustained fifth metatarsal fractures after our supplement trial ended. A cross-sectional study of professional basketball players supports a significant relationship between history of stress fractures and *highe*r vitamin D levels [29]. However, outside of basketball players, the literature remains mixed with regard to vitamin D and fracture risks in young, active, populations, as one report suggested that stress fractures were associated with lower (<40 ng/mL) 25(OH)D levels in soldiers [43] while another demonstrated no relationship between injury and serum 25-OH vitamin D levels in swimmers and divers [20]. Any potential disadvantage of vitamin D supplementation on bone health and fracture risk in basketball players thereby warrants further exploration.

### 4.4. Limitations

We acknowledge the limitations of extrapolating our largely negative results to a broader population, due to our small sample size (N = 18), modest weekly dosage (4000IU), short intervention period (12-weeks), highly specialized population (collegiate basketball players), and mix of ethnicities and genders within group analyses. However, we hope these data can launch future investigations involving larger cohorts of competitive athletes, which are historically difficult to study. To justify our small cohort, it is important to note that the number of basketball players per team remains limited (10–15/team), with small cohort numbers representative of the sport in general [1,26,30]. Additionally, we acknowledge that higher dosages of vitamin D (>4000 IU/day) have been suggested to promote ergogenic benefits [1], especially in athletes who are deficient. However, excessive supplementation (>70,000 IU/week) has also been shown to be detrimental [44]. We chose this conservative dosage because it reflects the current guidelines for healthy individuals [3; 22] and the maximum dosage we could reliably dispense within our Institutions’ regulatory constraints for individuals with normal (mean) baseline levels (>30 ng/mL) of 25(OH)D. Also, the dietary intakes of vitamin D, in our cohort of basketball players, were below recommended thresholds (~200 IU/day) but similar to a previous cross-sectional study performed on 41 collegiate athletes [7]. Increased dietary intake of vitamin D was positively associated with increased 25(OH)D at baseline (pre-intervention) in our athletes, demonstrating a potential confounding effect of not controlling vitamin D in the athletes’ diet during the intervention period.

We also recognize a few methodological limitations, which may have biased these results: 1) we did not verify the vitamin D_3_ dosage (4000IU) dispensed weekly, through an outside laboratory, and 2) we should have performed a block randomization to address the racial disparities within our (odd/even) randomization process, as duly performed in a previous study [16].

## 5. Conclusions

Modest Vitamin D_3_ supplementation did not elicit statistically significant or meaningful changes in body composition or performance (peak power) over placebo supplementation, in our cohort of collegiate basketball players. However, on an individual basis, players with the lowest 25(OH)D demonstrate the highest increases in 25(OH)D after modest supplementation. Thus, from a practical standpoint, targeted vitamin D_3_ supplementation should be encouraged in vitamin D insufficient or deficient basketball players to maintain adequate 25(OH)D levels throughout the year for health (rather than performance) reasons.

Additionally, from secondary and tertiary study perspectives, African American players demonstrated significantly lower vitamin D and greater fat mass losses compared with Caucasian players over 12 weeks of strength training. Lastly, from our exploratory study, the influence of sweat sodium (rather than calcium) losses on bone mass, at rest and during exercise, warrants further investigation as does the potential for enhanced fracture risks in basketball players given vitamin D supplements.

## Data Availability

The data that support the findings of this study are available from the corresponding author upon reasonable request
